# Overconfidence in the Cognitive Reflection Test: Comparing Confidence Resolution for Reasoning vs. General Knowledge

**DOI:** 10.3390/jintelligence11050081

**Published:** 2023-04-27

**Authors:** André Mata

**Affiliations:** CICPSI, Faculdade de Psicologia, Universidade de Lisboa, 1649-013 Lisboa, Portugal; aomata@psicologia.ulisboa.pt

**Keywords:** cognitive reflection, overconfidence, error monitoring, conflict detection

## Abstract

This research examines the metacognitive awareness that people have about their reasoning performance in the Cognitive Reflection Test (CRT). The first two studies compare confidence judgments about the CRT vs. general knowledge (GK) questions. Results show that (1) people are generally able to discriminate between correct and incorrect answers, but this ability is far from perfect, and it is greater for GK questions than for CRT problems. Indeed, and strikingly, (2) incorrect responses to CRT problems are produced with approximately the same level of confidence as *correct* responses to GK questions. However, (3) even though confidence is high for incorrect responses to CRT problems, it is even higher for correct responses. The results of two additional studies show that these differences in confidence are ultimately related to the conflict that CRT problems pose between intuition and deliberation. These findings have implications for the possibility of implicit error monitoring and dual-process models of overconfidence.

The Cognitive Reflection Test (CRT; Frederick 2005) has become quite popular in research on reasoning and behavioral economics (Bialek and Pennycook 2018; Branas-Garza et al. 2019). The problems in the test pose a conflict between an intuitive but incorrect response and a correct response that requires effortful deliberation. A good example is the well-known bat-and-ball problem: A bat and a ball together cost 1 dollar and 10 cents. The bat costs 1 dollar more than the ball. How much does the ball cost? The answer “10 cents” comes to mind immediately, but it is incorrect (the correct answer is 5 cents). The fact that the incorrect answer is so intuitive makes it likely (1) that many people fall for it, and (2) that they are highly confident in those answers, that is, that they are overconfident (e.g., Mata et al. 2013b; Pennycook et al. 2017). 

A key question in research on reasoning and metacognition is whether people who make reasoning errors are aware that their thinking is flawed. Incorrect responses to the CRT are often associated with great confidence. For instance, incorrect responders are willing to bet on the accuracy of their responses, they believe that most people would give the same responses as they did, and they tend to make high confidence ratings (e.g., De Neys et al. 2013; Johnson et al. 2016; Mata 2019a, 2020; Mata et al. 2013b). This would suggest that incorrect responders are completely unaware of the biased nature of their responses. 

Other studies, however, suggest that incorrect responders are aware, at least to some extent, of the biased nature of their reasoning. In particular, conflict detection studies (e.g., De Neys et al. 2011, 2013) show that reasoners react differently to conflict problems and no-conflict control versions of those problems (e.g., they take longer or are less confident for the former than the latter). Once again using the bat-and-ball problem as an example, it is easy to modify it (by eliminating the “more than the ball” part of the second premise) in order to remove the conflict: A bat and a ball together cost 1 dollar and 10 cents. The bat costs 1 dollar. How much does the ball cost? Now the intuitive response “10 cents” is correct. Because of this, these control problems produce virtually no errors, and the few participants who make those errors are removed from the analysis (e.g., De Neys et al. 2013). 

The present research takes a different approach to exploring overconfidence and error awareness in reasoning: Instead of (or in addition to—see Study 4) using control problems where intuition is correct, the studies presented here use a new type of control problems where there is no intuitive response, and for which participants must rely solely on deliberation. This type of control has a key advantage: It produces errors. That is, responses to these problems can be hard, just as for conflict problems, but unlike conflict problems, they are not tricky, as intuition is mute. This enables a novel approach to assessing the role of intuition and deliberation in overconfidence. Moreover, it is important to measure confidence for errors on such non-tricky questions, as they provide a relevant baseline against which to gauge error awareness in reasoning. Research on error monitoring for conflict reasoning problems (e.g., De Neys et al. 2013; Mata et al. 2013b) has thus far only examined confidence for three types of responses: Correct responses to tricky problems, incorrect responses to tricky problems, and correct responses to non-tricky problems. The missing cell—incorrect responses to non-tricky problems—enables two things: (1) A relevant baseline against which to measure the confidence in errors for conflict reasoning problems; and (2) a measure of resolution, that is, the degree to which responders discriminate between correct and incorrect responses, and whether that differs for conflict problems such as those included in the CRT vs. the no-conflict control problems introduced in these studies. 

Participants in the present studies responded to tricky conflict questions such as the bat-and-ball problem (i.e., questions for which intuition and deliberation suggest different responses), as well as non-tricky no-conflict questions (i.e., questions for which there is no intuition, and a person must rely on deliberation alone). Whereas Studies 3–4 manipulate conflict within the same domain (i.e., control versions of conflict problems were created), Studies 1–2 manipulate conflict across domains: Confidence was compared for CRT problems (i.e., conflict) vs. typical (i.e., no-conflict) general knowledge (GK) questions, which also represents a new approach in comparison to previous research. 

General knowledge is a domain where there has been extensive research on overconfidence. Here too people often show overconfidence (e.g., Atir et al. 2015; Fischhoff et al. 1977; Moore and Healy 2008). Critically, though, typical GK questions do not pose a conflict between intuition and deliberation. People either know the correct response (by deliberatively thinking about it), or they do not, but unlike CRT-type problems, GK questions are not designed to trigger (faulty) intuitions. As a consequence, even though in both domains people are known to be overconfident, confidence resolution should be higher for GK questions than for reasoning problems. That is, for GK questions, people should know better when they know the answer and when they do not, whereas, for CRT-type problems, they should be misled into trusting their intuitive but incorrect response. In other words, still, the double curse in the Dunning–Kruger effect (i.e., people who lack knowledge or competence in some area also lack awareness of those shortcomings; Dunning 2011; Kruger and Dunning 1999) should be greater when a problem triggers a misleading intuition than when that is not the case. 

For correct responses, on the other hand, confidence should be greater for CRT-type problems than for GK questions. This is because arriving at a correct response in tricky reasoning problems presumably implies overcoming the intuitive incorrect response. Thus, there is a two-step reasoning process, whereby the reasoner first thinks of the intuitive response and only later comes to the correct response (though I should note that the logical intuition framework would not predict this two-step process—for a review, see De Neys 2012; for more on this topic, see Section 5). This awareness of having overcome the intuitive but incorrect response and having solved a tricky problem should give correct reasoners a confidence boost (Mata 2019a; Mata et al. 2013b). 

For all studies, confidence ratings were collected for the four cells described above. Several comparisons are of theoretical relevance:

Comparing resolution (i.e., the difference in confidence for correct vs. incorrect responses) for CRT-type problems vs. GK questions shows whether the ability to tell correct responses from incorrect ones differs across domains. Conflict detection studies (e.g., De Neys et al. 2011, 2013) test whether responders react differently to conflict problems vs. control no-conflict problems, but they are not informative about how good their resolution is. The present studies test people’s ability to distinguish between correct and incorrect responses in cognitive reflection, as compared to their ability to monitor their knowledge in other domains.

(a) Comparing confidence for correct responses in conflict vs. no-conflict problems provides a test of the metacognitive advantage of having awareness of alternative solutions. According to the metacognitive advantage model (Mata 2019a, 2019b, 2020; Mata and Almeida 2014; Mata et al. 2013b), correct responders should have a confidence boost for conflict problems, as they realize that they were able to overcome a tricky problem for which there is a compelling but misleading response. For no-conflict problems, on the other hand, it is less likely that there is such an awareness of alternative responses, and therefore confidence for correct responses should be high, but not so high as for conflict problems.
(b) Comparing confidence for incorrect responses in conflict vs. no-conflict problems provides a test of whether reasoners can detect their reasoning errors to the same extent when there is an intuitive (though incorrect) response that comes to mind vs. when intuition is absent and responders rely solely on deliberation.
(c) Finally, comparing confidence for incorrect responses in conflict problems vs. confidence for *correct* responses to no-conflict problems provides an extreme test of failure to detect errors. Because tricky conflict problems are designed to trigger an intuitive (though incorrect) response, the confidence that comes with that response should be greater than the confidence that comes with errors in the no-conflict problems. Still, if reasoners realize somehow that they made a mistake, they should rate their confidence lower than that for correct responses to the control problems. If they do not, this is revealing of their awareness (or lack thereof) of their errors.

## 1. Study 1

### 1.1. Method

#### 1.1.1. Participants

Sixty participants, students at the University of Heidelberg, were recruited to take part in this study; fifty-nine completed the study. 

#### 1.1.2. Procedure

Participants completed the CRT, as well as a GK quiz (for all the studies, the task was computerized). The order in which participants completed the CRT and the quiz was counterbalanced across subjects. Within each block of problems (CRT or GK), the order of items was randomized. 

The GK quiz was a questionnaire used in research on overconfidence (Moore and Healy 2008). It contained 10 questions on several topics (see Appendix A). Responses were open-ended.

After each CRT or GK question, participants rated how confident they were that their response was correct, on a scale from 1: “not at all confident” to 9: “very confident”. After they responded to all the questions in a test (CRT or GK), they estimated how many of the X questions that they had just seen (3 for the CRT; 10 for GK) they answered correctly. I shall refer to these measures as local confidence and global confidence, respectively (Liberman 2004).

### 1.2. Results 

#### 1.2.1. Performance 

On average, participants gave 1.86 (*SD* = 1.06) correct responses for the CRT, and 6.64 (*SD* = 2.42) for GK. In percentage, there is no difference in performance across tests, *t* < 1. This eliminates a potential source of differences in overconfidence across domains: Difficulty (Kruger 1999; Pulford and Colman 1997; Schraw and Roedel 1994).

#### 1.2.2. Global Confidence

Comparing the estimated number of correct responses against that actual number provides a measure of over- or under-estimation. In the quiz, participants estimated that they were correct for 5.92 of the 10 questions (*SD* = 2.44), which is close to the actual number of correct responses (6.64), but significantly lower (i.e., under-estimation), *t*(58) = −2.92, *p* = .005, *d* = 0.38. For CRT problems, there was over-estimation: Participants estimated that they were correct for 2.41 of the 3 problems (*SD* = 0.65), which is significantly higher than the actual number of correct responses (1.86), *t*(58) = 4.65, *p* < .001, *d* = 0.61. Comparing these estimation errors for CRT problems vs. GK questions, there is a significant difference, *t*(58) = 5.85, *p* < .001, *d* = 0.76. Not only are these errors in estimation different in quality (i.e., over- vs. under-estimation), but they are also different in quantity: Comparing the absolute difference from estimated to actual correct responses shows that the miscalibration was greater for the CRT than for GK, *t*(58) = 2.05, *p* = .045, *d* = 0.27. 

#### 1.2.3. Local Confidence

Mean confidence ratings were compared for correct and incorrect responses to each kind of question (see Table 1).

The analyses presented here include both an ANOVA comparing confidence for correct and incorrect responses for both types of problem, as well as *t*-tests for specific comparisons of theoretical relevance, which were laid out in the Introduction (moreover, the ANOVA sacrifices several data points, as it requires that participants give all four types of response, whereas *t*-tests comparing only two cells preserve more data).

Paired-sample *t* tests show that (1) correct CRT responses were given with greater confidence than incorrect CRT responses, *t*(29) = 2.58, *p* = .015, *d* = 0.47; (2) correct GK responses were also associated with greater confidence than incorrect GK responses, *t*(45) = 8.38, *p* < .001, *d* = 1.24; (3) correct CRT responses were given with greater confidence than correct GK responses, *t*(50) = 4.95, *p* < .001, *d* = 0.69; (4) incorrect CRT responses were associated with greater confidence than incorrect GK responses, *t*(30) = 4.55, *p* < .001, *d* = 0.82; (5) indeed, confidence for incorrect CRT responses was above the midpoint (5) of the scale (in a one-sample *t* test, *p* < .001; compare that with the confidence shown for incorrect GK responses, which is below the midpoint of the scale, *p* = .056); (6) in fact, incorrect CRT responses were associated with the same confidence levels as correct GK responses, *t* < 1 (note that degrees of freedom change for the different comparisons, as not all participants gave all four types of responses).

Considering those participants who provided all four types of response, an ANOVA reveals a main effect of type of problem, *F*(1, 23) = 25.48, *p* < .001, η_p_^2^ = .53, such that confidence was higher for the CRT than for the GK quiz; a main effect of accuracy, *F*(1, 23) = 31.84, *p* < .001, η_p_^2^ = .58, such that confidence was higher for correct responses than for incorrect ones; and an interaction effect, *F*(1, 23) = 4.97, *p* = .036, η_p_^2^ = .18, such that the difference in confidence for correct vs. incorrect responses was larger for the GK quiz than for the CRT. 

## 2. Study 2

The second study seeks to replicate the findings of Study 1 with a larger sample and a different GK test that (1) contains more questions, (2) is pre-tested for the specific subject population, and (3) has a different format: Multiple choice instead of open-ended. In Study 1, there was no overconfidence for the GK quiz in the global confidence measure. However, even though this result is not unusual in global confidence measures (Liberman 2004), the recognition exercise involved in multiple-choice tests might lead to greater overconfidence than the recall exercise that is called for in open-ended questions. Still, it is important to note that there were no differences in difficulty (as measured by performance) across tests in the first study. The second study again controls for difficulty. 

### 2.1. Method

#### 2.1.1. Participants

Ninety students from the University of Heidelberg took part in the study. 

#### 2.1.2. Procedure

Participants completed the CRT and a GK quiz. The order in which participants completed the CRT and the quiz was counterbalanced across subjects. Within each block of problems (CRT or GK), the order of items was randomized. 

The GK quiz was a questionnaire designed to study overconfidence in a German sample (Michailova and Katter 2014). It contained 18 questions from several domains (see Appendix A). For each question, three response options were presented for participants to choose from.

After each CRT or GK question, participants rated how confident they were that their response was correct, on a scale from 1: “not at all confident” to 9: “very confident” (local confidence). When all items in a test (CRT or GK) were completed, participants were asked to estimate how many of the X questions they had just seen (3 for the CRT; 18 for the GK quiz) they had answered correctly (global confidence). 

### 2.2. Results 

#### 2.2.1. Performance

On average, participants had 1.69 (*SD* = 1.13) correct responses in the CRT and 11.39 (*SD* = 1.93) in the GK quiz. In percentage, there is no significant difference in performance across tests, *t*(89) = 1.66, *p* = .101, *d* = 0.16. This again suggests that any potential differences in overconfidence across domains are not due to differences in difficulty.

#### 2.2.2. Global Confidence

On average, participants estimated that they were correct for 9.62 of the 18 GK questions (*SD* = 2.85), which is below the actual number of correct responses (*M* = 11.39), *t*(89) = 5.61, *p* < .001, *d* = 0.59. For the CRT, in contrast, there was over-estimation: Participants estimated that they were correct for 2.34 of the 3 problems (*SD* = 0.72), which is significantly higher than the actual number of correct responses (*M* = 1.69), *t*(88) = −6.11, *p* < .001, *d* = 0.59. Comparing these estimation errors for CRT problems vs. GK questions, there is a significant difference, *t*(88) = 7.83, *p* < .001, *d* = 0.83.

Once again, these estimation errors differ not only in quality (i.e., over- vs. under-estimation) but also in quantity: Comparing the absolute difference from estimated to actual correct responses in proportion to the total number of responses in a test shows that the miscalibration was greater for CRT (*M* = 25.66%, *SD* = 31.09) than for GK (*M* = 14.70%, *SD* = 12.62), *t*(88) = 2.89, *p* = .005. 

#### 2.2.3. Local Confidence 

Mean confidence ratings were compared for correct and incorrect responses for each kind of question (see Table 2).

Paired-sample *t*-tests revealed that (1) correct CRT responses were given with greater confidence than incorrect CRT responses, *t*(41) = 2.91, *p* = .006, *d* = 0.45; (2) correct GK responses were also associated with greater confidence than incorrect GK responses, *t*(89) = 14.94, *p* < .001, *d* = 0.83; (3) correct CRT responses were associated with greater confidence than correct GK responses, *t*(71) = 6.97, *p* < .001, *d* = 0.82; (4) incorrect CRT responses were associated with greater confidence than incorrect GK responses, *t*(59) = 7.52, *p* < .001, *d* = 0.97; (5) indeed, confidence for incorrect CRT responses was above the midpoint (5) of the scale (in a one-sample *t* test, *p* < .001), whereas confidence for incorrect GK responses was below the midpoint of the scale (*p* < .001); (6) in fact, incorrect CRT responses were given with the same confidence as correct GK responses, *t* < 1. 

An ANOVA comparing the confidence ratings of those participants who gave all four types of response revealed a main effect of type of problem, *F*(1, 41) = 53.82, *p* < .001, η_p_^2^ = .57, such that confidence was higher for the CRT than for the GK quiz; a main effect of type of responding, *F*(1, 41) = 53.61, *p* < .001, η_p_^2^ = .57, such that confidence in correct responses was higher than confidence in incorrect responses; and an interaction, *F*(1, 41) = 9.00, *p* = .005, η_p_^2^ =.18, such that the difference in confidence for correct vs. incorrect responses was greater for GK than for the CRT. 

## 3. Study 3

Study 3 sought to provide further evidence about the source of the overconfidence of biased reasoners: Conflict. Whereas the previous studies manipulated conflict across domains (CRT problems were tricky, whereas GK questions were not), Study 3 compares confidence for conflict vs. no-conflict problems within the same domain: Cognitive reflection. Specifically, this study compares confidence for the CRT problems used in the previous studies (i.e., conflict problems where intuition and deliberation are at odds) with confidence for problems that are structurally similar, but which do not pose a conflict between intuition and deliberation, as there is no intuitive response (i.e., there is no response that comes immediately to mind, and only via deliberative reasoning can a person come to a response). The main prediction is that, if the reason for the overconfidence of incorrect CRT responders is that they are tricked by the intuitive lure, then resolution (i.e., the difference in confidence for correct vs. incorrect responses) should be higher for problems where there is no intuition competing for the response (i.e., deliberation-only problems) than for conflict problems where intuition and deliberation are at odds, and this is because confidence for incorrect responses should be higher in the latter case, as participants are tricked by their intuition. 

To the best of my knowledge, this control (no lure) has not been used for CRT-type problems, which is also a contribution of this study. As explained in the Introduction, typical control problems in this research prompt a correct intuitive response. By eliminating the intuitive pull of the problems, the control used in the present study provides a test of how poor resolution in cognitive reflection relates to faulty intuition.

### 3.1. Method

#### 3.1.1. Participants

One hundred participants were recruited from the Prolific Academic platform for online studies (a total of 101 completed the task; 49.5% female, 50.5% male; *M* age = 33.15, *SD* = 9.34). The language was English.

#### 3.1.2. Procedure

Participants answered 3 CRT-type problems (the conflict problems) as in Studies 1–2, as well as 3 problems that were analogous to the conflict problems, but where there was no intuitive response (e.g., A cake and a piece of fruit cost $8.47 in total. The cake costs $6.71. How much does the piece of fruit cost?—see Appendix A). For each problem, participants made confidence ratings from 1: “not at all confident” to 9: “very confident” (global confidence was not assessed in this study). The order of the 6 problems was randomized.

### 3.2. Results 

#### 3.2.1. Performance

Even though the mathematics required to solve conflict problems is presumably easier than that required for the no-conflict/deliberation-only problems, the number of correct responses was lower for conflict vs. no-conflict problems, demonstrating the intuitive pull of conflict problems: *M* = 1.36, *SD* = 1.18 vs. *M* = 2.44, *SD* = 0.73, *t*(100) = 8.93, *p* < .001, *d* = 0.89.

#### 3.2.2. Confidence 

Confidence ratings are presented in Table 3 as a function of conflict and performance. Replicating the previous studies, (1) responders were more confident when they were correct vs. incorrect for conflict problems, *t*(42) = 4.64, *p* < .001, *d* = 0.71; (2) the same difference holds for no-conflict problems, *t*(42) = 4.60, *p* < .001, *d* = 0.70; (3) correct responses to conflict problems were produced with greater confidence than correct responses to no-conflict problems, *t*(67) = 2.72, *p* = .008, *d* = 0.33; (4) incorrect responses to conflict problems were presented with greater confidence than incorrect responses to no-conflict problems, though this difference was marginally significant, *t*(34) = 1.88, *p* = .068, *d* = 0.32, which most likely relates to the fact that this was the pairwise comparison (out of the 6 tests presented here) with fewer datapoints; indeed, the difference in overall values for these cells (not conditional on whether participants committed both types of error) is larger when one considers the values in Table 3 than when one considers the smaller range of cases where both types of error occurred; (5) confidence for incorrect responses to conflict problems was above the midpoint (5) of the scale (in a one-sample *t* test, *p* < .001, whereas confidence for incorrect responses to no-conflict problems was not, *p* = .386); and (6) finally, in this study, confidence for incorrect responses to conflict problems was lower than that expressed for correct responses to no-conflict problems, *t*(73) = -4.27, *p* < .001, *d* = 0.50. Still, confidence for incorrect responses to conflict problems was quite high (*M* = 6.27, on a 9-point scale). 

More importantly, and replicating the previous studies, resolution was lower for conflict vs. no-conflict problems: An ANOVA comparing the confidence ratings of those participants who gave all four responses revealed the predicted conflict-by-accuracy interaction, *F*(1, 14) = 4.90, *p* = .044, η_p_^2^ = .23, such that the difference in confidence for correct vs. incorrect responses was larger for no-conflict than for conflict problems (see Table 3); there was also a main effect of accuracy, *F*(1, 14) = 9.51, *p* = .008, η_p_^2^ = .40, such that confidence was higher for correct vs. incorrect responses; the main effect of conflict was not significant, *F* < 1.

## 4. Study 4

Study 4 seeks to replicate the results of the previous studies with a broader set of items that have been used in previous research on reasoning and general knowledge (adapted from Ackerman 2014; Boland 2013; Erickson and Mattson 1981; Fischhoff et al. 1977; Frederick 2005; Juslin et al. 2000; Koriat 1995, 2008; Mata et al. 2013a; Oldrati et al. 2016; Sirota et al. 2021; Thomson and Oppenheimer 2016; Van Dooren et al. 2005; Weber 2016). In particular, various problems tapping various thinking-and-reasoning domains were used in this study, including standard CRT-type items, verbal CRT problems (less dependent on mathematical reasoning), GK items, semantic illusions, and others (see Appendix A), so as to establish the robustness of the key finding of Studies 1–3 (the fact that resolution is lower for conflict vs- no-conflict problems) with a wide range of problems. Moreover, by using a larger set of problems as well as a larger sample, this study seeks to increase the number of participants who give all four responses (i.e., both correct and incorrect responses to conflict and no-conflict problems), which are necessary for the resolution analysis.

Critically, and as in Study 3, this study includes conflict and no-conflict versions of the same problems, in order to control for the critical factor presumed to have generated the difference in resolution for conflict vs. no-conflict items in the first studies: Conflict. Ultimately, there is nothing inherent to CRT problems and GK questions that makes the former necessarily trickier and therefore more prone to generate overconfidence than the latter. Indeed, CRT problems can be simple (e.g., the no-conflict control problems used in research on conflict detection and implicit error monitoring) and GK questions can be tricky (e.g., Erickson and Mattson 1981; Fischhoff et al. 1977; Juslin et al. 2000; Koriat 1995). To the extent that one domain is trickier than the other, it should produce greater overconfidence. The key hypothesis, again, is that the discrimination between good and bad performance (i.e., resolution) should be poorer for conflict tasks, which trigger an intuitive but incorrect response, than for no-conflict tasks, which do not trigger those faulty intuitions.

Finally, this study uses a different type of control problem: Whereas in Studies 1–3, no-conflict problems were such that deliberation was called for, as there was no intuitive response that was prompted by the problems, the problems used in this study trigger an intuitive response that is incorrect. This is the type of control problem that is used in research on cognitive reflection and conflict detection (e.g., De Neys et al. 2011, 2013; Mata 2020; Mata and Almeida 2014). The goal of using this type of control in this study is two-fold: First, it seeks to test whether the difference in resolution for conflict vs. no-conflict problems observed in the previous studies holds using the type of control problems typically used in this area of research. More importantly, by using no-conflict control problems that trigger an intuitive though incorrect response, this study enables an assessment of the automatic vs. controlled processes underlying overconfidence in cognitive reflection. Specifically, by using a large and balanced number of conflict and no-conflict problems, this study enables using the Process Dissociation Procedure (PDP; Jacoby 1991; Jacoby et al. 1993). This analytical method uses performance in conflict and no-conflict trials to disentangle the controlled (C) vs. automatic (A) components of the mental processing that goes into solving a cognitive task (see equations in the results section). This procedure has been successfully applied to both reasoning (Ferreira et al. 2006; Mata et al. 2013c) and general knowledge tasks (Mata et al. 2013a). The hypotheses are that the higher the A scores, the more compelling the intuitive responses will be, and therefore the more overconfident participants will be in their incorrect responses to conflict trials, and the less able they will be to distinguish between correct and incorrect responses. The opposite goes for the C parameter: The higher the ability to control the intuitive responses, the better able participants will be to know when they are correct vs. incorrect. In addition, this C parameter might explain the confidence boost that was observed in the previous studies when participants showed greater confidence for correct responses to conflict vs. no-conflict trials. That is, to the extent that conflict problems trigger tricky intuitions but correct responders are able to override them, they should be extra confident in their correct responses, as compared to their confidence in correct responses to no-conflict trials, which do not call for controlling one’s intuition. 

### 4.1. Method

#### 4.1.1. Participants

One hundred and forty undergraduates from the University of Lisbon took part in the study (79.1% female, 20.1% male, and one participant who did not indicate their gender; *M* age = 20.86, *SD* = 7.14). One participant gave nonsensical responses (random strings of letters) and was therefore not considered in the analysis. 

#### 4.1.2. Procedure

Participants answered 20 problems (the 3 CRT-type problems used in the previous studies, plus 17 new items; see Appendix A). Half of them were tricky conflict problems, which prompted an intuitive but incorrect response, whereas the other half were no-conflict problems, where the intuitive response was correct. There were two versions of the problems, counterbalanced across subjects, such that half of the participants saw a specific problem in its conflict version, whereas the other half saw it without conflict. Conflict problems were transformed into no-conflict versions by changing small details in the premises (see Appendix A). The order of the 20 items was randomized. 

As in the previous studies, after each problem/question, participants rated how confident they were that their response was correct, on a scale from 1: “not at all confident” to 9: “very confident”. 

After participants solved all the problems and made confidence ratings for each of them, they completed additional tasks, related to another project, irrelevant to the present purposes.

### 4.2. Results 

#### 4.2.1. Performance

The number of correct responses was lower for conflict vs. no-conflict problems: *M* = 3.19, *SD* = 1.75 vs. *M* = 8.36, *SD* = 1.40, *t*(138) = 27.89, *p* < .001, *d* = 2.37.

#### 4.2.2. Confidence

Paired-sample *t* tests revealed that (1) correct responses to conflict items were given with greater confidence than incorrect responses to those items, *t*(134) = 4.15, *p* < .001, *d* = 0.36; (2) correct responses to no-conflict items were also associated with greater confidence than incorrect responses to those items, *t*(106) = 13.34, *p* < .001, *d* = 1.29; (3) correct responses to conflict problems were produced with the same confidence as correct responses to no-conflict problems, *t*(134) = −1.31, *p* = .191, *d* = 0.12; (4) incorrect responses to conflict problems were associated with greater confidence than incorrect responses to no-conflict problems, *t*(106) = 9.63, *p* < .001, *d* = 0.93; (5) indeed, confidence for incorrect responses to conflict problems was above the midpoint (5) of the scale (in a one-sample *t* test, *p* < .001; compare that with the confidence shown for incorrect responses to no-conflict problems, which is below the midpoint of the scale, *p* < .001); (6) finally, incorrect responses to conflict problems were given with less confidence than correct responses to no-conflict problems, *t*(138) = −8.15, *p* < .001, *d* = 0.70. Still, and even though this last result suggests some error awareness, the mean confidence for incorrect responses to conflict problems was quite high. 

More importantly, and replicating all the previous studies, resolution was lower for conflict vs. no-conflict problems. An ANOVA comparing the confidence ratings of participants who gave all four responses revealed a main effect of conflict, *F*(1, 103) = 67.68, *p* < .001, η_p_^2^ = .40, such that confidence was actually higher for conflict problems than for no-conflict problems; a main effect of accuracy, *F*(1, 103) = 170.34, *p* < .001, η_p_^2^ = .62, such that confidence in correct responses was higher than confidence in incorrect responses; and the predicted conflict-by-accuracy interaction, *F*(1, 103) = 62.88, *p* < .001, η_p_^2^ = .38, such that the difference in confidence for correct vs. incorrect responses was greater for no-conflict than for conflict problems (see Table 4). 

#### 4.2.3. PDP

Finally, a PDP analysis was performed by calculating the C parameter as P(correct/non-conflict)-P(incorrect/conflict), and A as P(incorrect/conflict)/(1-C) (Ferreira et al. 2006, 2016; Mata et al. 2013c). After excluding negative C values (which are usually interpreted as resulting from random error and therefore excluded, e.g., Jacoby et al. 1993; Ferreira et al. 2006; Mata et al. 2013c), the C parameter predicts the difference in confidence for correct vs. incorrect responses to conflict problems (i.e., resolution for the critical conflict problems), *r* = .22, *p* = .019; the difference in confidence for correct responses to conflict problems vs. correct responses to no-conflict problems (i.e., the confidence boost when responding correctly to conflict problems), *r* = .19, *p* = .046; the difference in confidence for incorrect responses to conflict problems vs. incorrect responses to no-conflict problems, *r* = −.42, *p* < .001, such that the higher the C scores, the lower this difference (i.e., less overconfidence for errors in conflict vs. no-conflict problems); and the difference in resolution (i.e., the difference in mean confidence for correct vs. incorrect responses) for conflict vs. no-conflict problems, *r* = −.43, *p* < .001. Thus, those responders who were better able to control their intuitive responses were also better at knowing the difference between correct and incorrect responses and proved better able to tell right from wrong for both conflict and no-conflict trials. 

The A parameter has opposite effects for the most part (even though C and A are unrelated, *r* = .03, *p* = .752): It correlates negatively with resolution (i.e., the difference in confidence for correct vs. incorrect responses) for conflict trials, *r* = −.24, *p* = .011; it correlates negatively with the difference in confidence for correct responses to conflict vs. no-conflict trials, although this correlation was not significant, *r* = −.11, *p* = .251, suggesting a dissociation, whereby the confidence boost of correct conflict responders is only explained by the C parameter; the A parameter correlates positively with the difference in confidence for incorrect responses to conflict problems vs. incorrect responses to no-conflict problems, *r* = .29, *p* = .009, such that the higher the A scores, the more confidence responders had for errors in conflict vs. no-conflict problems; and it predicts the difference in resolution (i.e., the difference in mean confidence for correct vs. incorrect responses) for conflict vs. no-conflict problems, *r* = .25, *p* = .023, such that the more compelling the intuitive responses, the harder it was to tell right from wrong (i.e., resolution) for conflict trials, as compared to no-conflict trials.

## 5. General Discussion

Four studies with both online and lab samples from different nationalities (German, US, and Portuguese) assessed overconfidence in reasoning about the CRT and explored its origins. The results of Studies 1–2 were remarkably consistent: Participants showed some ability to discriminate between correct and incorrect answers, but this ability was greater for GK questions, whereas for CRT problems it was poorer. Indeed, the most striking result in these studies is that participants were as confident when they responded incorrectly to CRT problems as when they responded correctly to GK questions. However, even though incorrect responders were hugely (over)confident in their solutions to CRT problems, correct responders were even more confident. In fact, the confidence associated with correct responses was higher for CRT problems than for GK questions. Studies 3–4 provided evidence for the key factor underlying these results: Conflict. Specifically, overconfidence among incorrect responders is greater when problems trigger an intuitive but incorrect response. For those tricky items, people are less able to discriminate between correct and incorrect responses. 

### 5.1. Unskilled and Unaware: On Conflict Detection and Error Monitoring

The overconfidence of incorrect reasoners was patent in several results: Even though confidence for correct responses to conflict problems was higher than for errors in those problems, (1) this discrimination was lower for conflict problems than for no-conflict problems, (2) confidence for incorrect responses to conflict problems was above the midpoint of the scale, whereas for incorrect responses to no-conflict problems it was lower, and (3) it was sometimes as high for incorrect responses to conflict problems as for correct responses to no-conflict problems (in Studies 1–2, though not in Studies 3–4). Altogether, these results suggest that incorrect reasoners do not have a very clear sense of their errors.

This is inconsistent with research on conflict detection (De Neys et al. 2011, 2013), which suggests that incorrect responders are aware of their reasoning shortcomings, as they react differently to different versions of a problem that vary in whether intuition is in conflict or in harmony with deliberation. For instance, reasoners have been shown to be less confident when providing incorrect responses to conflict problems than when they respond correctly to no-conflict problems (e.g., De Neys et al. 2011, 2013; see also Mata 2020). 

Despite the evidence for conflict sensitivity when comparing metacognitive reactions across different versions of a problem, one can question whether that qualifies as cogent evidence for true error detection, given how high the confidence ratings usually are for incorrect responses to reasoning problems. Indeed, one question that conflict detection studies evoke is what the criteria for error detection should be, and whether comparing participants’ reactions to conflict vs. no-conflict versions is the only type of evidence this research should be concerned with. If participants realize at some level that their responses are incorrect, should they not express low (and not just lower) confidence levels? However, the confidence levels of incorrect reasoners are typically above the midpoint of the scale, as in the present studies, and sometimes as high as 85% or even 91.5% (where 100% means completely confident; De Neys et al. 2013; Johnson et al. 2016). 

Thus, the metacognitive judgments that people make for conflict problems should tell us something about whether they are truly aware of their reasoning shortcomings. One thing is for people to detect that there is something different in conflict vs. no-conflict problems. A different thing is to assume that, because of such differences, people realize that they are wrong. Underlying such differences might be true sensitivity to logical principles (for compelling evidence that conflict detection is related to knowledge of logical principles, see Burič and Šrol 2020; Frey et al. 2017; Šrol and De Neys 2021), but sometimes it might be mere sensitivity to peripheral features of a problem, unrelated to logic (Aczel et al. 2016; Ferreira et al. 2022; Ghasemi et al. 2023, 2022; Klauer and Singmann 2013; Mata et al. 2014; Meyer-Grant et al. 2022).

Therefore, the possibility of implicit conflict detection is fascinating, and the amount of evidence supporting it is impressive, but there is a long way from conflict sensitivity to error awareness, and one should be cautious in inferring the latter from the former. Several recent developments are being made in this area of research, which will help to make sense of conflict detection results and what they mean for the possibility of implicit error monitoring (e.g., Šrol and De Neys 2021).

The evidence suggests that the overconfidence of incorrect responders originates from their being generally unaware of alternative responses. For instance, when participants in a study by Mata et al. (2013b) were made aware of the correct alternative response (presented as a possible response; not necessarily the correct response), they dramatically lowered their confidence in the incorrect responses that they had given earlier. Similarly, when participants were asked, after having given their responses, to think of alternative responses that other people might give to the same problem, they grew less confident in their original solutions (Mata 2019a, 2019b). Moreover, studies using measures of decisional conflict, where participants are asked whether they considered alternative responses and how divided they felt between different possible responses before committing to a final response, also show that the overconfidence of incorrect responders is related to being oblivious to alternative responses (Mata et al. 2013b; Mata 2019a, 2020). Thus, past research suggests that confidence is related to reasoners’ (un)awareness of alternative responses. 

The present Study 4 directly tested this mechanism by manipulating whether the problems presented to participants pose a conflict between intuition or deliberation, i.e., whether the problems prompt an intuitive (but incorrect) response, or not. As predicted, the overconfidence of incorrect responders was greatest when problems brought to mind an intuitive but incorrect response. Moreover, the PDP analysis in Study 4 suggests that the more compelling this intuitive response was (as measured by the A parameter), the less able incorrect responders were to discriminate between correct and incorrect responses, particularly for tricky conflict problems. Thus, the present studies add to previous research by shedding light on the mechanisms underlying the overconfidence of biased reasoners. 

### 5.2. Skilled and Aware: The Metacognitive Advantage of Deliberative Responders

These results largely replicate the results of Mata et al. (2013b) in that correct responses to tricky reasoning problems were given with great confidence. Indeed, comparing confidence in correct responses to conflict vs. no-conflict problems shows that the latter were high, but the former was even higher (in Studies 1–3, though not in Study 4). Thus, whereas incorrect responders to conflict problems are overconfident, correct responders can be said to be *superconfident*. I suggest that the reason for this confidence boost is the awareness that the correct responders have of the intuitive but incorrect response. Presumably, they too thought of that response before they came to the correct solution (Mata 2019a, 2020; Šćeta et al. 2022). This awareness that the problem is tricky, but that they were able to override their initial misleading intuition, gives them great confidence in their response. 

Several of the studies previously referenced already provide some evidence for this mechanism, either manipulating or measuring whether reasoners consider alternative responses (e.g., Mata 2019a). The present Study 4 adds to those studies in that the PDP analysis provides new evidence for the metacognitive advantage model: To the extent that participants were better able to control and override the intuitive (but incorrect) responses to conflict problems (as measured by the C parameter), they had a confidence boost in their correct responses (as compared to the confidence they showed for their correct responses to no-conflict versions of the same problems). 

The present studies were not designed to test the hypotheses with response time analysis (admittedly, a limitation). Indeed, because the conflict and no-conflict problems were often of different lengths (and in one study, they were also of different formats: Open-ended for CRT vs. multiple choice for GK), a response time analysis might not be very rigorous or informative. However, were such an analysis feasible using comparable versions of the problems, one might consider what the prediction would be. On the one hand, previous research shows that correctly solving conflict problems can be more time-consuming than doing so for non-conflict problems (e.g., Bonner and Newell 2010; Ferreira et al. 2016; Stupple et al. 2011), which is compatible with a default-interventionist dual-process perspective whereby correct responses can only be produced after overcoming faulty intuitions. However, other research shows that the relation between response time and accuracy is not as straightforward as such a perspective would predict (Stupple et al. 2017), and some studies suggest that correct responses can be intuitive, with no need for effortful deliberation (e.g., Bago and De Neys 2017). Most likely, the reality is more complicated than simply assuming that correct responding always or never requires deliberation. Future research should continue to pursue the fine-grained exploration of individual differences (and their causes) in the degree to which accuracy is related to effortful deliberation or logical intuitions (Burič and Šrol 2020; Frey et al. 2017; Stupple et al. 2011).

At this point, one might ask the question of when conflict (i.e., having two answers in mind) boosts vs. decreases confidence. The key moderating factor, I believe, is whether there is a verifiable correct response to the problem or not (Trouche et al. 2014). When such a response exists, as is the case for reasoning problems, one can think about it and check that it is correct. Together with being aware of the intuitive but incorrect response, this boosts the confidence of the correct responder (Mata 2019a, 2020; Mata et al. 2013b). However, when there is no objectively correct response, as in the case of moral dilemmas, having competing responses in mind might undermine confidence. There is evidence for this in studies showing that conflict decreases perceptions of consensus, such that those who feel more divided about how to respond to moral dilemmas believe that fewer people share their responses (Mata 2019b). Future studies might test whether the existence (vs. lack) of a response that can be demonstrated to be objectively correct (Trouche et al. 2019) determines whether response conflict and revision boost confidence or rather undermines it.

In conclusion, this metacognitive mechanism whereby confidence judgments are informed by the awareness (or lack thereof) of alternative responses underlies both the curse of the incorrect responders (Dunning 2011) and the advantage of the correct responders (Mata 2019a, 2020; Mata et al. 2013b). Because incorrect responders do not realize that there are other, more valid, possible responses, they do not know the correct response, and they do not know that they do not know. Correct responders, on the other hand, realize that there are alternative solutions that are tempting but incorrect, and so they know, and they know that they know.

## Figures and Tables

**Table 1:** Mean confidence ratings (and SD) by accuracy and type of problem (Study 1).

	CRT	GK
Correct	8.49 (1.32)	7.11 (1.45)
Incorrect	6.82 (2.23)	4.35 (2.23)

**Table 2:** Mean confidence ratings (and SD) by accuracy and type of problem (Study 2).

	CRT	GK
Correct	7.99 (1.74)	6.72 (1.05)
Incorrect	6.56 (2.04)	4.09 (1.58)

**Table 3:** Mean confidence ratings (and SD) by accuracy and type of problem (Study 3).

	Conflict	No-Conflict
Correct	8.05 (1.49)	7.33 (1.59)
Incorrect	6.27 (2.17)	5.37 (2.81)

**Table 4:** Mean confidence ratings (and SD) by accuracy and type of problem (Study 4).

	Conflict	No-Conflict
Correct	6.86 (1.67)	7.05 (0.99)
Incorrect	6.27 (1.36)	4.17 (2.26)

## Data Availability

Data will be provided upon request.

## Appendix A

Reasoning problems used in Studies 1–2:

A bat and a ball together cost 110 cents. The bat costs 100 cents more than the ball. How much does the ball cost?

If it takes 5 machines 5 min to make 5 widgets, how long would it take 100 machines to make 100 widgets?

In a lake, there is a patch of lily pads. Every day, the patch doubles in size. If it takes 48 days for the patch to cover the entire lake, how long would it take for the patch to cover half of the lake?

GK questions used in Study 1:

Antibiotics kill what class of pathogen?

What is the largest species of whale?

Who wrote and directed “Kill Bill” Volumes 1 and 2?

What was the full name of the cannibalistic main character in the film “Silence of the Lambs”?

Who painted the Sistine Chapel?

The Italian village of Pompeii was destroyed in 79 AD by what type of natural disaster?

Jim Morrison (lead singer of The Doors), Elvis Presley, and Jimi Hendrix all died from what?

What was the name of the famous 3-day music festival, held in 1969, which featured artists such as Bob Dylan, Janis Joplin, and Jimi Hendrix?

What is the name of the world’s largest coral reef, located off the coast of Australia?

What is the highest mountain range in the world?

**Table A1:** GK questions used in Study 2.

Questions	Response Options		
What is the name for an instant camera?	Canon camera	Polaroid camera	Minolta camera
Where do flounders usually live?	Among the reeds	Amongst coral reefs	On the sea bottom
What is a rollmop made of?	herring	pork	salmon
What country does the Nobel Prize winner in Literature Gabriel García Márquez come from?	Spain	Venezuela	Colombia
What artistic movement does anacreontics belong to?	Rococo	Romanticism	Realism
What is a hot chilli sauce?	Tabasco	Curacao	Macao
How many letters are there in the Russian alphabet?	40	33	26
“Tosca” is an opera by …?	G. Puccini	G. Verdi	A. Vivaldi
What is the name of the Greek Goddess of wisdom?	Pallas Athena	Nike	Penelope
What is the most abundant metal on Earth?	iron	Aluminum	Copper
What is a word to describe an unknowing person?	Ignatius	ignorant	ideologue
Who was the first person to fly around the Eiffel Tower in an airship?	Santos-Dumont	Count Zeppelin	Saint-Exupéry
What is the name of Eskimo snow shelter?	wigwam	igloo	tipi
What enterprise belongs to Bill Gates?	Intel	Microsoft	Dell Computers
What is the Islamic month of fasting called?	Sharia	Ramadan	Imam
What language does the term “Fata Morgana” come from?	Arabic	Swahili	Italian
How long does it take for a hen to hatch an egg?	21 days	14 days	28 days
What is ascorbic acid?	apple vinegar	vitamin A	vitamin C

**Table A2:** Problems used in Study 3.

Conflict Version	No-Conflict Version
A TV and a DVD together cost 88 euros. The TV costs 80 euros more than the DVD. How much does the DVD cost?	A cake and a piece of fruit cost $8.47 in total. The cake costs $6.71. How much does the piece of fruit cost?
If it takes 10 hens 10 days to lay 10 eggs, how long would it take 100 hens to lay 100 eggs?	If it takes 14 min for a turtle to lay 5 eggs, how long would it take for the turtle to lay 100 eggs?
A computer virus is spreading through the system of a computer. Every minute, the number of infected files doubles. If it takes 100 min for the virus to infect all of the system, how long would it take for the virus to infect half of the system?	In a town, there is a flood. If it takes 48 days for the water to cover the entire town, how long would it take for it to cover a town that is 5 times larger?

**Table A3:** Problems used in Study 4.

Conflict Version (Intuitive and Correct Responses, and Source)	No-Conflict Version
A TV and a DVD together cost 88 euros. The TV costs 80 euros more than the DVD. How much does the DVD cost? (intuitive response = 8; correct response = 4; Mata and Almeida 2014, adapted from Frederick 2005)	A TV and a DVD together cost 88 euros. The TV costs 80 euros. How much does the DVD cost?
If it takes 10 hens 10 days to lay 10 eggs, how long would it take 100 hens to lay 100 eggs? (intuitive response = 100; correct response = 10; Mata and Almeida 2014, adapted from Frederick 2005)	If 10 hens lay 10 eggs in one day, how many eggs would 100 chickens lay in one day?
A computer virus is spreading through the system of a computer. Every minute, the number of infected files doubles. If it takes 100 min for the virus to infect all of the system, how long would it take for the virus to infect half of the system? (intuitive response = 50; correct response = 99; Mata and Almeida 2014, adapted from Frederick 2005)	A computer virus is spreading through the system of a computer. Every minute, one file gets infected. If it takes 100 min for the virus to infect all of the system, how long would it take for the virus to infect half of the system?
A farmer had 15 sheep and all but 8 died. How many are left? (intuitive response = 7; correct response = 8; Thomson and Oppenheimer 2016)	A farmer had 15 sheep and 8 died. How many are left?
How many cubic feet of dirt are there in a hole that is 3’deep x 3’ wide x 3’ long? (intuitive response = 27; correct response = none; Thomson and Oppenheimer 2016)	How many cubic feet of water are there in a fish tank that is 3’deep x 3’ wide x 3’ long?
Steve was standing in a long line. To amuse himself he counted the people waiting, and saw that he stood 15th from the beginning and 15th from the end of the line. How many people stood in the line? (intuitive responses = 31 or 30; correct response = 29; Ackerman 2014)	Steve was standing in a long line. To amuse himself he counted the people waiting, and saw that there were 15 people in front of him and 15 behind him. How many people stood in the line?
Ellen and Kim are running around a track. They run equally fast but Ellen started later. When Ellen has run 5 laps, Kim has run 15 laps. When Ellen had run 30 laps, how many has Kim run? (intuitive response = 90; correct response = 40; Van Dooren et al. 2005)	Ellen and Kim are running around a track. Kim runs 3 times as fast as Ellen. When Ellen has run 5 laps, Kim has run 15 laps. When Ellen had run 30 laps, how many has Kim run?
One month of the year has 28 days. How many of the remaining 11 months have 30 days? (intuitive response = 5; correct response = 11; Weber 2016);	One month of the year has 28 days. How many of the remaining 11 months have 31 days?
You have a book of matches and enter a cold, dark room. You know that in the room there is an oil lamp, a candle, and a heater. What do you light first? (intuitive response = oil lamp, candle, or heater; correct response = matches; Boland 2013)	You enter a cold, dark room. You know that in the room there is an oil lamp, a candle, and a heater. What do you light first?
There are 25 soldiers in a row standing 3 m from each other. How long is the row? (intuitive response = 75 m; correct response = 72 m; Oldrati et al. 2016).	There are 25 vans in a row. Each of them is 3 m long. How long is the row?
Which city is further north: Rome or New York? (intuitive response = New York; correct response = Rome; Juslin et al. 2000)	Which city is further north: Rio de Janeiro or New York?
What is the capital of California: San Francisco, Los Angeles, or Sacramento? (intuitive responses = San Francisco, Los Angeles; correct response = Sacramento; Koriat 1995)	What is the largest city in California: San Francisco, Los Angeles, or Sacramento?
Would it be ethical for a man to marry the sister of his widow? (intuitive response = yes or no, justified; correct response = impossible, he is dead; Sirota et al. 2021)	Would it be ethical for a man to marry the sister of his wife?
The wind blows west. An electric train runs east. In which cardinal direction does the smoke from the locomotive blow? (intuitive response = west; correct response = there is no smoke in an electric train; Sirota et al. 2021)	The wind blows west. A train runs east. In which cardinal direction does the smoke from the locomotive blow?
What is the capital of Australia: Sydney, Canberra, or Melbourne? (intuitive response = Sidney; correct response = Canberra; Weber 2016)	What is the largest city in Australia: Sydney, Canberra, or Melbourne?
Who was the Mathematician who was the author of the theorem that states that the square of the length of the hypotenuse of a rectangle is equal to the sum of squares of the lengths of other sides? (intuitive response = Pythagoras; correct response = no one; it was a triangle, not a rectangle; Mata et al. 2013a)	Who was the Mathematician who was the author of the theorem that states that the square of the length of the hypotenuse of a triangle is equal to the sum of squares of the lengths of other sides?
When was the first air raid: 1849 or 1937? (intuitive response = 1937; correct response = 1849; Fischhoff et al. 1977; Koriat 2008)	When was the first air raid: 1819 or 1849?
How many animals of each kind did Moses take in the ark? (intuitive response = 2; correct response = zero; Erickson and Mattson 1981)	How many animals of each kind did Noah take in the ark?
What are the names of the Portuguese archipelagos located to the east of continental Portugal? (intuitive response = Madeira and Azores; correct response = the archipelagos are located to the west, not east; Mata et al. 2013a)	What are the names of the Portuguese archipelagos located to the west of continental Portugal?
Potatoes are native to Ireland or Peru? (intuitive response = Ireland; correct response = Peru; Fischhoff et al. 1977)	Potatoes are native to Peru or Japan?

## References

[B1-jintelligence-11-00081] Ackerman Rakefet (2014). The diminishing criterion model for metacognitive regulation of time investment. Journal of Experimental Psychology: General.

[B2-jintelligence-11-00081] Aczel Balazs, Szollosi Aba, Bago Bence (2016). Lax monitoring versus logical intuition: The determinants of confidence in conjunction fallacy. Thinking & Reasoning.

[B3-jintelligence-11-00081] Atir Stav, Rosenzweig Emily, Dunning David (2015). When knowledge knows no bounds: Self-perceived expertise predicts claims of impossible knowledge. Psychological Science.

[B4-jintelligence-11-00081] Bago Bence, De Neys Wim (2017). Fast logic?: Examining the time course assumption of dual process theory. Cognition.

[B5-jintelligence-11-00081] Bialek Michal, Pennycook Gordon (2018). The cognitive reflection test is robust to multiple exposures. Behavior Research Methods.

[B6-jintelligence-11-00081] Boland Sam (2013). Reflection expanded: Expanding the Cognitive Reflection Task. https://pt.scribd.com/document/138024235/Reflection-Expanded-Expanding-the-Cognitive-Reflection-Task.

[B7-jintelligence-11-00081] Bonner Carissa, Newell Ben R. (2010). In conflict with ourselves? An investigation of heuristic and analytic processes in decision making. Memory & Cognition.

[B8-jintelligence-11-00081] Branas-Garza Pablo, Kujal Praveen, Lenkei Balint (2019). Cognitive reflection test: Whom, how, when. Journal of Behavioral and Experimental Economics.

[B9-jintelligence-11-00081] Burič Roman, Šrol Jakub (2020). Individual differences in logical intuitions on reasoning problems presented under two-response paradigm. Journal of Cognitive Psychology.

[B10-jintelligence-11-00081] De Neys Wim (2012). Bias and conflict: A case for logical intuitions. Perspectives on Psychological Science.

[B11-jintelligence-11-00081] De Neys Wim, Rossi Sandrine, Houdé Olivier (2013). Bats, balls, and substitution sensitivity: Cognitive misers are no happy fools. Psychonomic Bulletin & Review.

[B12-jintelligence-11-00081] De Neys Wim, Cromheeke Sofie, Osman Magda (2011). Biased but in doubt: Conflict and decision confidence. PLoS ONE.

[B13-jintelligence-11-00081] Dunning David (2011). The Dunning–Kruger effect: On being ignorant of one’s own ignorance. Advances in Experimental Social Psychology.

[B14-jintelligence-11-00081] Erickson Thomas D., Mattson Mark E. (1981). From words to meaning: A semantic illusion. Journal of Memory and Language.

[B15-jintelligence-11-00081] Ferreira Mário B., Mata André, Donkin Christopher, Sherman Steven J., Ihmels Max (2016). Analytic and heuristic processes in the detection and resolution of conflict. Memory & Cognition.

[B16-jintelligence-11-00081] Ferreira Mário. B., Soro Jerônimo C., Reis Joana, Mata André, Thompson Valerie A. (2022). When Type 2 Processing Misfires: The Indiscriminate Use of Statistical Thinking about Reasoning Problems. Journal of Intelligence.

[B17-jintelligence-11-00081] Ferreira Mário B., Garcia-Marques Leonel, Sherman Steven J., Sherman Jeffrey W. (2006). Automatic and controlled components of judgment and decision making. Journal of Personality and Social Psychology.

[B18-jintelligence-11-00081] Fischhoff Baruch, Slovic Paul, Lichtenstein Sarah (1977). Knowing with certainty: The appropriateness of extreme confidence. Journal of Experimental Psychology: Human Perception and Performance.

[B19-jintelligence-11-00081] Frederick Shane (2005). Cognitive reflection and decision making. Journal of Economic Perspectives.

[B20-jintelligence-11-00081] Frey Darren, Johnson Eric D., Neys Wim De (2017). Individual differences in conflict detection during reasoning. The Quarterly Journal of Experimental Psychology.

[B21-jintelligence-11-00081] Ghasemi Omid, Handley Simon, Howarth Stephanie, Newman Ian R., Thompson Valerie A. (2022). Logical intuition is not really about logic. Journal of Experimental Psychology: General.

[B22-jintelligence-11-00081] Ghasemi Omid, Handley Simon. J., Howarth Stephanie (2023). Illusory intuitive inferences: Matching heuristics explain logical intuitions. Cognition.

[B23-jintelligence-11-00081] Jacoby Larry L. (1991). A process dissociation framework: Separating automatic from intentional uses of memory. Journal of Memory and Language.

[B24-jintelligence-11-00081] Jacoby Larry L., Toth Jeffrey P., Yonelinas Andrew P. (1993). Separating conscious and unconscious influences of memory: Measuring recollection. Journal of Experimental Psychology: General.

[B25-jintelligence-11-00081] Johnson Eric D., Tubau Elisabet, Neys Wim De (2016). The Doubting System 1: Evidence for automatic substitution sensitivity. Acta Psychologica.

[B26-jintelligence-11-00081] Juslin Peter, Winman Anders, Olsson Henrik (2000). Naive empiricism and dogmatism in confidence research: A critical examination of the hard–easy effect. Psychological Review.

[B27-jintelligence-11-00081] Klauer Karl Christoph, Singmann Henrik (2013). Does logic feel good? Testing for intuitive detection of logicality in syllogistic reasoning. Journal of Experimental Psychology: Learning, Memory, and Cognition.

[B28-jintelligence-11-00081] Koriat Asher (1995). Dissociating knowing and the feeling of knowing: Further evidence for the accessibility model. Journal of Experimental Psychology: General.

[B29-jintelligence-11-00081] Koriat Asher (2008). Subjective confidence in one’s answers: The consensuality principle. Journal of Experimental Psychology: Learning, Memory, and Cognition.

[B30-jintelligence-11-00081] Kruger Justin (1999). Lake Wobegon be gone! The “below-average effect” and the egocentric nature of comparative ability judgments. Journal of Personality and Social Psychology.

[B31-jintelligence-11-00081] Kruger Justin, Dunning David (1999). Unskilled and unaware of it: How difficulties in recognizing one’s own incompetence lead to inflated self-assessments. Journal of Personality and Social Psychology.

[B32-jintelligence-11-00081] Liberman Varda (2004). Local and global judgments of confidence. Journal of Experimental Psychology: Learning, Memory, and Cognition.

[B33-jintelligence-11-00081] Mata André (2019a). Further tests of the metacognitive advantage model: Counterfactuals, confidence and affect. Psychological Topics (Special Issue on Meta-Reasoning).

[B34-jintelligence-11-00081] Mata André (2019b). Social metacognition in moral judgment: Decisional conflict promotes perspective taking. Journal of Personality and Social Psychology.

[B35-jintelligence-11-00081] Mata André (2020). Metacognition and social perception: Bringing meta-reasoning and social cognition together. Thinking and Reasoning.

[B36-jintelligence-11-00081] Mata André, Almeida Tiago (2014). Using metacognitive cues to infer others’ thinking. Judgment and Decision Making.

[B37-jintelligence-11-00081] Mata André, Schubert Anna-Lena, Ferreira Mário B. (2014). The role of language comprehension in reasoning: How “good enough” representations induce biases. Cognition.

[B38-jintelligence-11-00081] Mata André, Fiedler Klaus, Ferreira Mário B., Almeida Tiago (2013a). Reasoning about others’ reasoning. Journal of Experimental Social Psychology.

[B39-jintelligence-11-00081] Mata André, Ferreira Mário B., Reis Joana (2013b). A process-dissociation analysis of semantic illusions. Acta Psychologica.

[B40-jintelligence-11-00081] Mata André, Ferreira Mário B., Sherman Steven J. (2013c). The metacognitive advantage of deliberative thinkers: A dual-process perspective on overconfidence. Journal of Personality and Social Psychology.

[B41-jintelligence-11-00081] Meyer-Grant Constatin G., Cruz Nicole, Singmann Henrik, Winiger Samuel, Goswami Spriha, Hayes Brett K., Klauer Karl Christoph (2022). Are logical intuitions only make-believe? Reexamining the logic-liking effect. Journal of Experimental Psychology: Learning, Memory, and Cognition.

[B42-jintelligence-11-00081] Michailova Julija, Katter Joana K. (2014). Quantifying overconfidence in experimental finance. International Journal of Behavioural Accounting and Finance.

[B43-jintelligence-11-00081] Moore Don, Healy Paul J. (2008). The trouble with overconfidence. Psychological Review.

[B44-jintelligence-11-00081] Oldrati Viola, Patricelli Jessica, Colombo Barbara, Antonietti Alessandro (2016). The role of dorsolateral prefrontal cortex in inhibition mechanism: A study on cognitive reflection test and similar tasks through neuromodulation. Neuropsychologia.

[B45-jintelligence-11-00081] Pennycook Gordon, Ross Robert M., Koehler Derek J., Fugelsang Jonathan A. (2017). Dunning–Kruger effects in reasoning: Theoretical implications of the failure to recognize incompetence. Psychonomic Bulletin & Review.

[B46-jintelligence-11-00081] Pulford Briony D., Colman Andrew M. (1997). Overconfidence: Feedback and item difficulty effects. Personality and Individual Differences.

[B47-jintelligence-11-00081] Šćeta Lamija, Sliško Josip, Erceg Nikola (2022). Predicting the most common incorrect response: Metacognitive advantage of deliberative over intuitive responders on Cognitive Reflection Test. Studia Psychologica.

[B48-jintelligence-11-00081] Schraw Gregory, Roedel Teresa Debacker (1994). Test difficulty and judgment bias. Memory & Cognition.

[B49-jintelligence-11-00081] Sirota Miroslav, Drewberry Chris, Juanchich Marie, Valus Lenka, Marshall Amanda C. (2021). Measuring Cognitive Reflection without Maths: Developing and Validation of the Verbal Cognitive Reflection Test. Journal of Behavioral Decision Making.

[B50-jintelligence-11-00081] Šrol Jakub, De Neys Wim (2021). Predicting individual differences in conflict detection and bias susceptibility during reasoning. Thinking and Reasoning.

[B51-jintelligence-11-00081] Stupple Edward J., Ball Linden J., Evans Jonathan S. B., Kamal-Smith Emily (2011). When logic and belief collide: Individual differences in reasoning times support a selective processing model. Journal of Cognitive Psychology.

[B52-jintelligence-11-00081] Stupple Edward J., Pitchford Melanie, Ball Linden J., Hunt Thomas E., Steel Richard (2017). Slower is not always better: Response-time evidence clarifies the limited role of miserly information processing in the Cognitive Reflection Test. PLoS ONE.

[B53-jintelligence-11-00081] Thomson Kella S., Oppenheimer Daniel M. (2016). Investigating an alternate form of the cognitive reflection test. Judgment and Decision Making.

[B54-jintelligence-11-00081] Trouche Emmanuel, Sander Emmanuel, Mercier Hugo (2014). Arguments, more than confidence, explain the good performance of reasoning groups. Journal of Experimental Psychology: General.

[B55-jintelligence-11-00081] Trouche Emmanuel, Shao Jing, Mercier Hugo (2019). Objective evaluation of demonstrative arguments. Argumentation.

[B56-jintelligence-11-00081] Van Dooren Wim, Bock Dirk De, Hessels An, Janssens Dirk, Verschaffel Lieven (2005). Not everything is proportional: Effects of age and problem type on propensities for overgeneralization. Cognition and Instruction.

[B57-jintelligence-11-00081] Weber Mel (2016). The Role of Metacognition and Anxiety in College Students’ Performance on a General-Knowledge Test (Honors Thesis). https://pdfs.semanticscholar.org/7ae5/ee960a080a62fe15cd9e17fe01265ec1e522.pdf?_ga=2.29673148.2064039678.1582733636-1459196501.1522233394.

